# Primary dynamization versus standard static interlocking nailing in treatment of tibial shaft fractures in adults: a randomized controlled trial

**DOI:** 10.1186/s12891-025-09376-3

**Published:** 2025-12-12

**Authors:** Moustafa Elsayed, Abdelrahman Hafez Khalefa, Mohamed Salah Hafez Mazen, Khalaf Fathy Elsayed Ahmed

**Affiliations:** Orthopaedics and Traumatology Department, Sohag Faculty of Medicine, Sohag University, Sohag, Egypt

**Keywords:** Interlocking nail, Tibial shaft fractures, Primary dynamization, Without proximal locking, Randomized controlled trial

## Abstract

**Background:**

Interlocking nail (ILN) is gold standard for fixation of tibial shaft fractures in adults. Delayed union and non-union of tibial shaft fractures requiring secondary dynamization are high even with modern ILN techniques. There is no consensus to which dynamization technique has better outcomes and fewer complications. The purpose of this randomised controlled trial (RCT) was comparison of functional and radiological outcomes of primary dynamization without proximal locking screws versus standard static ILN in treatment of middle or distal thirds tibial shaft fractures in adults.

**Methods:**

This RCT study was conducted on 40 adult patients with middle or distal third tibial shaft fractures, treated by primary dynamization ILN without proximal locking (group A) or standard static ILN (group B), at orthopedics department of Sohag university hospital, between September 2022 and September 2024. Primary outcomes were rate of union, time to union, and incidence of complications. Secondary outcomes were Bostman knee score, American Orthopaedic Foot and Ankle Society (AOFAS) ankle-hindfoot score, and visual analog score (VAS).

**Results:**

Mean ages of patients at time of operation were 33.25 years for group A and 31.45years for group B. Duration of surgery in group A was 66.25 ± 4.83 min compared to group B 75 ± 5.38 min, *(P =* .044*)*. The mechanism of injury in most of patients was road traffic accident. The mean time to complete radiographic union in group A was 14 ± 1.86 weeks compared to group B at 16 ± 1.86 weeks, *(P <* .001*)*. Bostman knee score was higher in group A at 28.05 ± 2.33 points compared to group B at 26.1 ± 2.65 points, *(P =* .016*)*. No statistically significant difference was found between the two groups with respect to AOFAS ankle-hindfoot score, VAS for pain, rate of union or incidence of complications, *(P >* .05*)*.

**Conclusion:**

Both techniques achieved complete bony union and pain relief with minor complications. Primary dynamization of ILN tibia without proximal locking screws had advantages of shorter duration of surgery, faster radiographic complete union, and better functional outcomes.

**Level of evidence:**

Level II: Randomised controlled trial.

**Trial registration:**

The trial was retrospectively registered at 19 November, 2023 at www.clinicaltrials.gov (Trial Registry no: NCT06147219).

**Supplementary Information:**

The online version contains supplementary material available at 10.1186/s12891-025-09376-3.

## Background

Tibial shaft fractures in adults are one of the most common long bone fractures. They frequently originate from high energy injuries and make up 2% of all adult fractures [1, 2].

The primary goal of surgical management of tibial shaft fractures is to achieve complete bony union as early as possible to allow for rapid weight-bearing, early functional recovery and quick return to work. Interlocking nailing (ILN) by different surgical approaches was established as the gold standard technique for fixation of closed and most of open tibial shaft fractures in adults [3–5]. However, non-union rates in tibial shaft fractures were estimated to be 4.6–8.6% after modern intramedullary nailing techniques for closed fractures with much higher rates for open fractures [6, 7].

The socioeconomic burdens for tibial shaft non-unions and delayed unions are increasing including both direct (the treatment itself) and indirect (losses of productivity with prolonged treatment duration) costs [8, 9].

Dynamization of ILN is a surgical tool to promote fracture healing by compressing of and increasing contact between bone fragments at the fracture site. However, there is no consensus among different authors regarding either the optimal technique of dynamization or its timing for better outcomes. Different techniques for dynamization of ILN have been proposed; (1) Primary dynamization: fixation of ILN in a dynamic mode (interlocking screws are implanted in the oblong screw holes in a position that allows interlocking nail to move relative to them and compression of the fracture site), (2) Secondary dynamization of primary static ILN in cases of non-union or delayed union by removal of the farthest screw(s) from the fracture site (with either screw preservation in the oblong (dynamic) hole or removal of all screws at one nail end), and (3) Self dynamization: locking screw breakage (however it is a complication) may induce progression of fracture healing [10–13].

Unfortunately; the union rates after the current available dynamization techniques for tibial shaft fracture are estimated to be only 54–95.8% [14].

The purpose of this randomised controlled trial (RCT) was comparison of functional and radiological outcomes of primary dynamization ILN without proximal locking screws versus standard static ILN in treatment of middle or distal third tibial shaft fractures in adults.

Our hypothesis was that primary dynamization of ILN without proximal locking screws could show comparable outcomes to standard static ILN in treatment of middle or distal third tibial shaft fractures in adults as the proximal locking screws of ILN tibia weren’t needed if adequate rotational stability could be obtained intra-operatively, and fractures didn’t involve the proximal third. This improves the time to union and functional activities, simplifies the surgical procedure, and reduces the duration of surgery, fluoroscopy times and need for secondary interventions.

## Methods

### Trial design

Parallel-group, non-inferiority randomized controlled trial (RCT) was performed at the orthopedics and trauma department of Sohag university hospital, from September 2022 to September 2024. Approval for the study was obtained from our institution’s Medical Ethical Committee of Sohag faculty of medicine (IRB no: Soh-Med-23-010−04). It was registered retrospectively at 19 November, 2023 at ClinicalTrials.gov (NCT06147219).

This study was done according to the declarations of Helsinki, and informed written consents with risks explanation were obtained from all participants. The CONSORT guidelines for reporting RCTs were followed [15] (Supplementary file 1).

### Sample size calculation

A post hoc power analysis was performed using the TTestIndPower function from the statsmodels statistical package to evaluate the achieved power of the study. Assuming a two-sided significance level of 0.05, a sample size of 40 patients (20 patients per group), and a moderate to large effect size (Cohen’s d = 0.8), the estimated statistical power was 70.3%. While this falls slightly below the conventional threshold of 80%, it still provides reasonable confidence in detecting clinically meaningful differences between the study groups.

### Participants (inclusion and exclusion criteria)

The inclusion criteria of this study were: adult patients (18–50 years) with recent unilateral displaced middle third or distal third tibial shaft fractures of AO types (42-A, 42-B1, or 42-B2) [16], simple or open fractures (Gustilo-Anderson grades I) [17]. The exclusion criteria were; patients (< 18 or > 50 years), fractures of AO types (42-B3, or 42-C), open fractures (Gustilo-Anderson grade II or III), proximal third tibial shaft fractures, neglected old fractures (> 14 days), non-displaced fractures, intra-articular extension of fractures, pathological fractures, osteoporosis, sclerotic or deformed bone.

### Randomization

#### Sequence generation

An independent biostatistician who wasn’t involved in patient clinical care or outcomes evaluation created the random allocation sequence. To assign the participants, a computer-generated random number table was utilized (allocation ratio 1:1) either to group A: (Primary dynamization ILN) or group B: (Standard static ILN).

#### Allocation concealment mechanism

The random distribution details were kept in sequentially numbered, opaque, sealed envelopes prepared by the same independent biostatistician. This method ensured allocation concealment and prevented prior knowledge of group assignments.

#### Implementation

A research coordinator who didn’t participate in the surgical procedures or outcomes evaluation was responsible for enrolling the participants. The coordinator opened the subsequent consecutive envelope to identify the assigned intervention after enrollment and verification of eligibility. To maintain allocation concealment till the point of intervention, surgeons were only informed of the allocation after envelope was opened. All surgeries were done by surgeons qualified with both techniques.

### Blinding

It was difficult to blind the surgeons or participants to the treatment type because of open-labeled nature of study as both intervention techniques were visibly different. Thus, no blinding was used during the intervention phase, which is typical for surgical trials where the type of surgery cannot be concealed.

### Interventions and surgical techniques

#### Pre-operative assessment

At presentation to the emergency department medical history was taken and examination of patients for detection of associated injuries was done for each patient. Local examination of the injured leg for side of injury, swelling, signs of compartment syndrome, and neurovascular status was done.

All patients were evaluated by plain radiographs in the form of antero-posterior (AP), and lateral views of whole fractured leg including ipsilateral knee and ankle joints. Necessary pre-operative laboratory investigations were done for all patients. All fractures were classified using the AO/OTA classification system [16, 18]. Open fractures were classified by Gustilo–Anderson system [19].

#### Surgical techniques

Operations were done once general condition was stable. Patient was positioned supine on radiolucent operating table with roller rest for knee of affected side. Prophylactic intravenous antibiotics were administered 30 min prior to skin incision. Operations were performed with spinal anaesthesia and guided by C-arm. Thigh tourniquet was applied. Both K.F.E.A. and M.S.H.M. conducted the surgeries for both groups to avoid surgical skill bias. All patients were fixed with titanium tibia interlocking nail (Orthomed E^®^, Giza, Egypt).

**Fig. 1:**
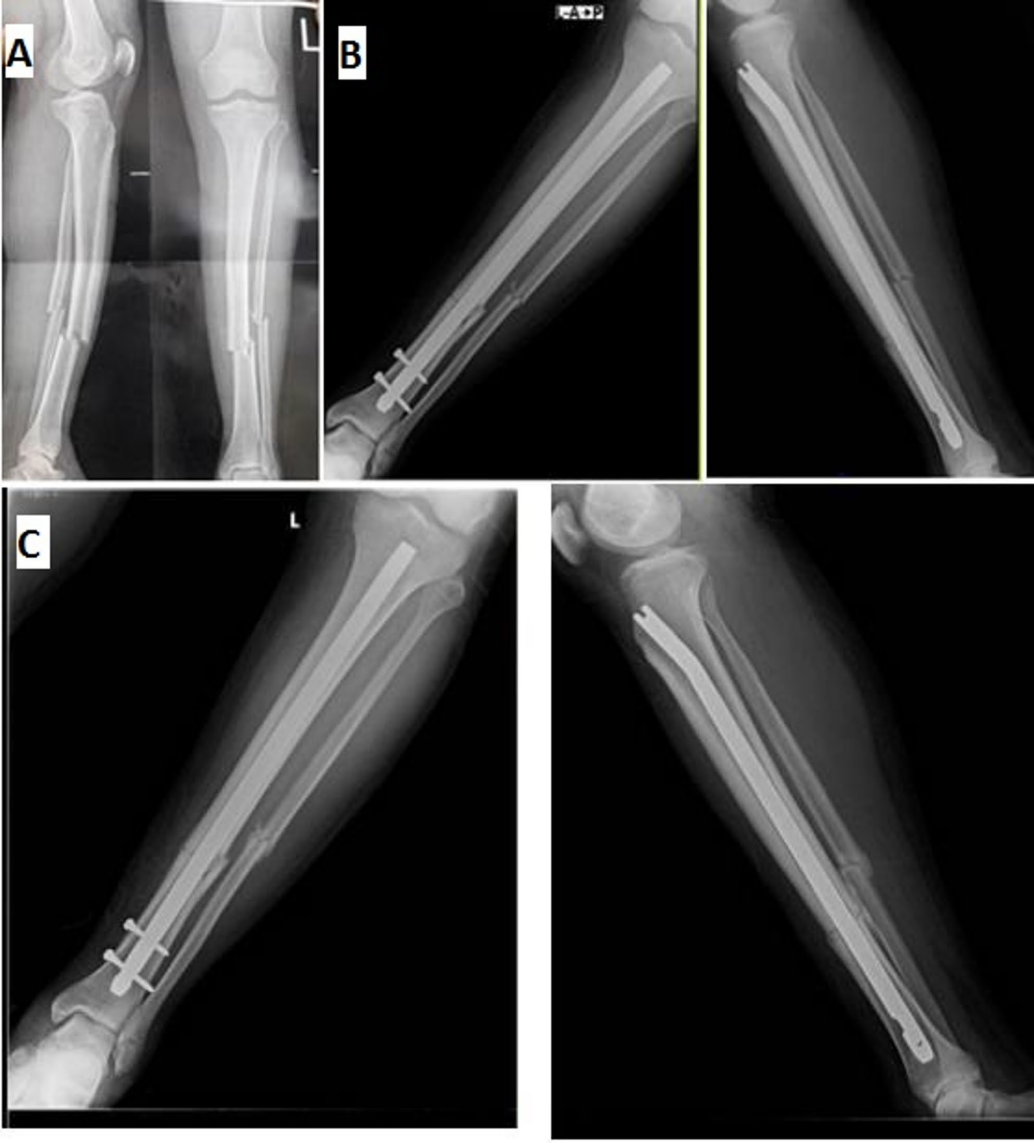
Surgical technique of primary dynamization ILN without use of proximal locking screws: **A** Pre-operative AP and lateral left leg radiographs of a 32 years old male patient showing tibial shaft fracture at the junction of middle and lower thirds, AO type 42-A3. **B** Immediate post-operative radiographs. **C** 4th week post-operative follow up radiographs

**Fig. 2:**
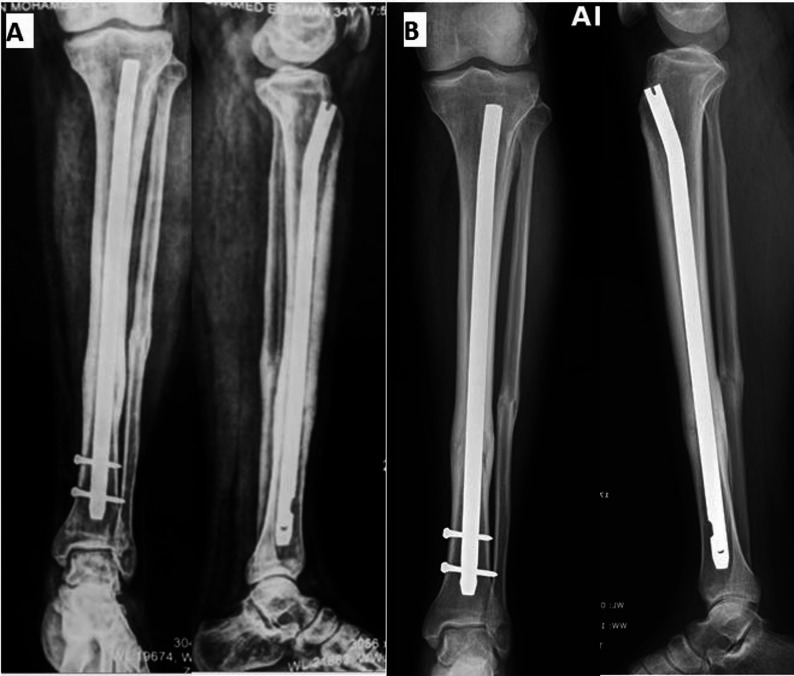
Surgical technique of primary dynamization ILN without use of proximal locking screws (continued): **A** follow up radiographs at the 4th month showing complete bony union. **B** One year follow up radiographs
B) Standard static ILN technique:
  The same technique was applied as primary dynamization nailing with addition of proximal locking screws in a static mode (Fig. 3).

A) Primary dynamization ILN without proximal locking screws technique:
  Longitudinal midline skin incision was made at the knee extending from lower pole of patella to 3 cm distally, then splitting of patellar tendon was made. The entry point of the nail was made with a curved awl under C-arm guidance. It was located in a line with intra-medullary canal of tibia just medial to lateral tibial spine in AP view, and just distal to the angle between the articular tibial plateau and anterior tibial metaphysis in lateral view. Ball-tipped guide wire was introduced from entry point till reaching the fracture site. Fracture was reduced manually by longitudinal traction to correct translation, angulation, and restoration of length. Reduction tools as pointed forceps may be used if needed. Then, guide wire was passed through fracture site till reaching the distal metaphysis within 1 cm from joint line, centered in AP and lateral views. Medullary canal reaming with sharp reamers starting from 7 mm reamer with 0.5 mm incremental diameters was performed till cortical chatter occurred to allow use of largest possible nail diameter with press-fit impaction in the medullary cavity. Nail diameter of 1 mm smaller than the last reamer was chosen. Length of nail was determined using a ruler under C-Arm. Ball-tipped guide wire was replaced with another guide wire over a plastic tube. The nail was attached to the jig and inserted in the canal over the guide wire. Then, guide wire was removed. The proximal end of the nail was kept within 0.5 to 2.0 cm from the subchondral bone. Two distal locking screws were inserted in a static mode by free-hand technique after “perfect rounded holes” were obtained. No proximal locking screws were used. Rotational stability was tested by passive mobilization of the operated leg, the intramedullary nail was considered stable if the leg and nail system moved as one piece on visual and fluoroscopic assessment and absence of distraction at the fracture site (Figs. 1 and 2).

**Fig. 3:**
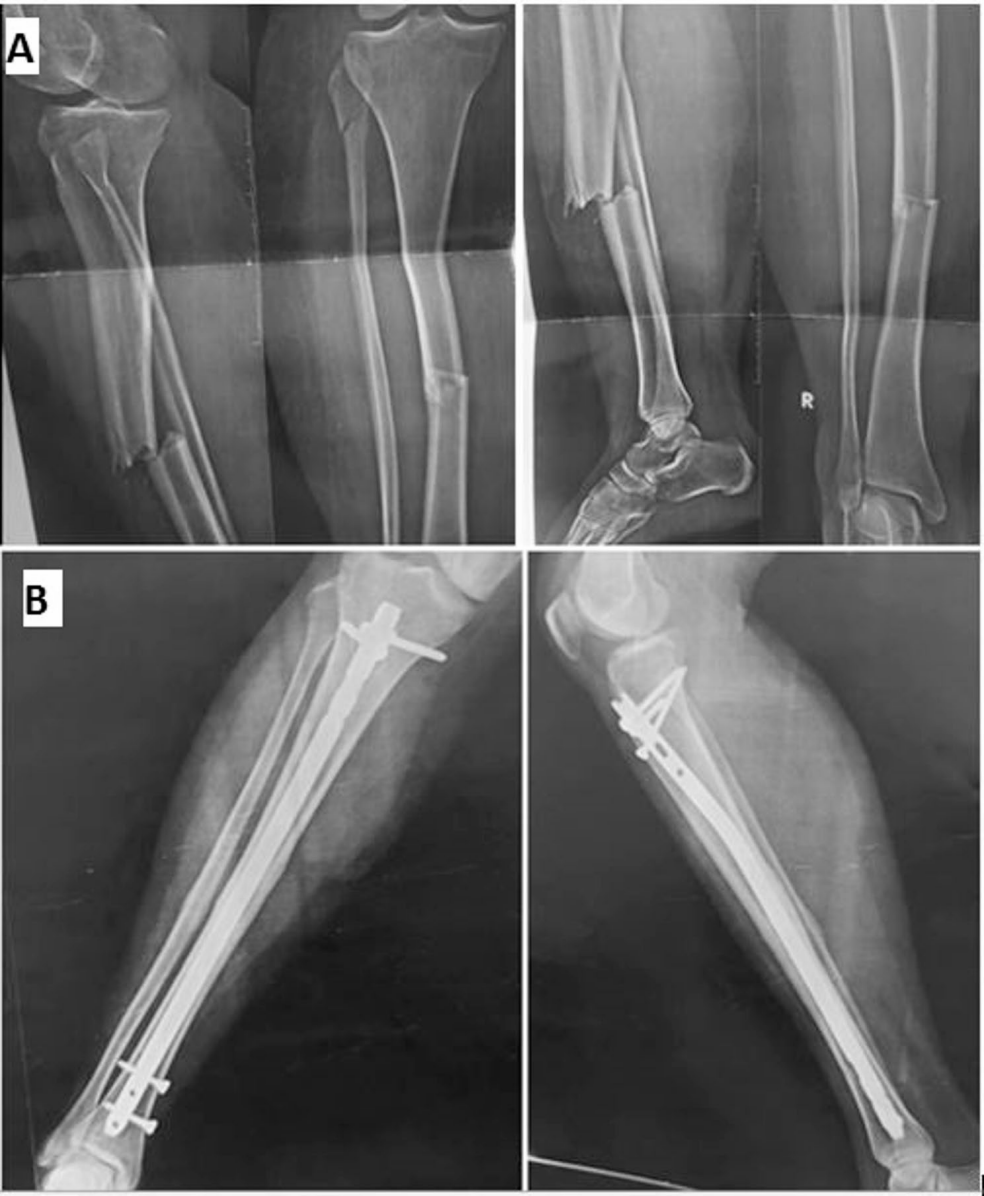
Surgical technique of standard static ILN: **A** Pre-operative AP and lateral right leg radiographs of a 30 years old female patient showing middle third tibial shaft fracture, AO type 42-A3. **B** Follow up radiographs at the 4th month showing complete bony union

### Post-operative care

Immediate postoperative AP and lateral plain radiographic views of whole fractured leg including ipsilateral knee and ankle joints were done for evaluation of fracture reduction and fixation.

The same combination of antibiotics which was used prophylactically before surgery was used for 48 h postoperatively in standard dose. Anti-inflammatory, anti-edematous, and anti-coagulant drugs were given to all patients. Patients were monitored for compartment syndrome. After 2 weeks stitches were removed.

Quadriceps exercises and partial-weight bearing were started soon when the patient was comfortable and according to the tolerance of the patient. Full weight-bearing was started when fractures healed.

### Outcomes assessment

1. Patients were followed up at our hospital clinic at: 2, 4, 6, and 8 weeks then at monthly interval till 12 months, then at yearly intervals. Patients were evaluated clinically and radiographically by two authors (M.E. and A.H.K.) who were blinded to the surgical technique.
  (a) Clinical evaluation was done for pain, ability of weight bearing, ROM of knee and ankle joints, and any complication.
  (b) Radiographic evaluation of patients by AP and lateral plain x-ray views of the whole leg including ipsilateral knee and ankle at monthly interval till fracture union and then at 6 monthly intervals for one year for assessment of fracture reduction, bone union, and any complications.
2. Radiographic union was evaluated by modified radiographic union scale for tibial fractures (mRUST) Score [20]. The mRUST score considers four cortices (anterior, posterior, medial, and lateral) on two views (AP and lateral) of the tibia. Each cortex is scored from 1 to 4, based on the quality of healing observed (1 = no callus and fracture line visible, 2 = callus present and fracture line visible, 3 = bridging callus and fracture line visible, and 4 = remodeled, fracture line not visible). The scores of all cortices were then combined to give a total score (4–16); minimum score of 4 (definitely not healed) and a maximum of 16 (completely healed). No or minimal sign of union at end of 6 months was considered as non-union. The total mRUST score of ≥ 13 in addition to absence of tenderness at the fracture site on palpation is indicative of fracture union.
3. Functional outcomes scores as Visual Analogue Scale (VAS) for pain intensity, Bostman knee score, and AOFAS ankle-hindfoot score were determined at the end of 1 st year [21–23]. Bostman knee score was calculated by giving the patient points according to range of motion (ROM, 3–6 points), pain (0–6 points), working ability (0–4 points), quadriceps femoris atrophy (0–4 points), walking with assistance (0–4 points), effusion (0–2 points), giving way (0–2 points), and climbing stair (0–2 points). The maximum total score was 30 points: a score of 30–28 points was excellent, 20–27 points was good, and less than 20 points was fair. VAS (0 represents “no pain”, 1–3 “Moderate pain”, 4–9 “Severe pain” while 10 represents “the worst pain imaginable”). AOFAS ankle-hind foot score consists of maximum 100 points; 40 for pain, 50 for function, and 10 points for alignment. The results were classified as excellent (90–100), good (75–89), fair (50–74), and Poor (< 50).

## Statistical analysis

Analysis of data was performed with the IBM-SPSS 27.0 software. Testing of normality of data was done by using the Kolmogorov-Smirnov test and Shapiro-Wilks test. Qualitative variables were described by numbers and percentages (N, %), whereas quantitative variables described by mean ± standard deviation (SD). Chi-square test and fisher exact test were used to compare qualitative variables between the two groups when appropriate, whereas comparing quantitative variables between the two groups was done by Student’s t-test for parametric variables, and Mann–Whitney U test for non-parametric variables. A two-tailed *p* <.05 was considered statistically significant.

## Results

**Fig. 4:**
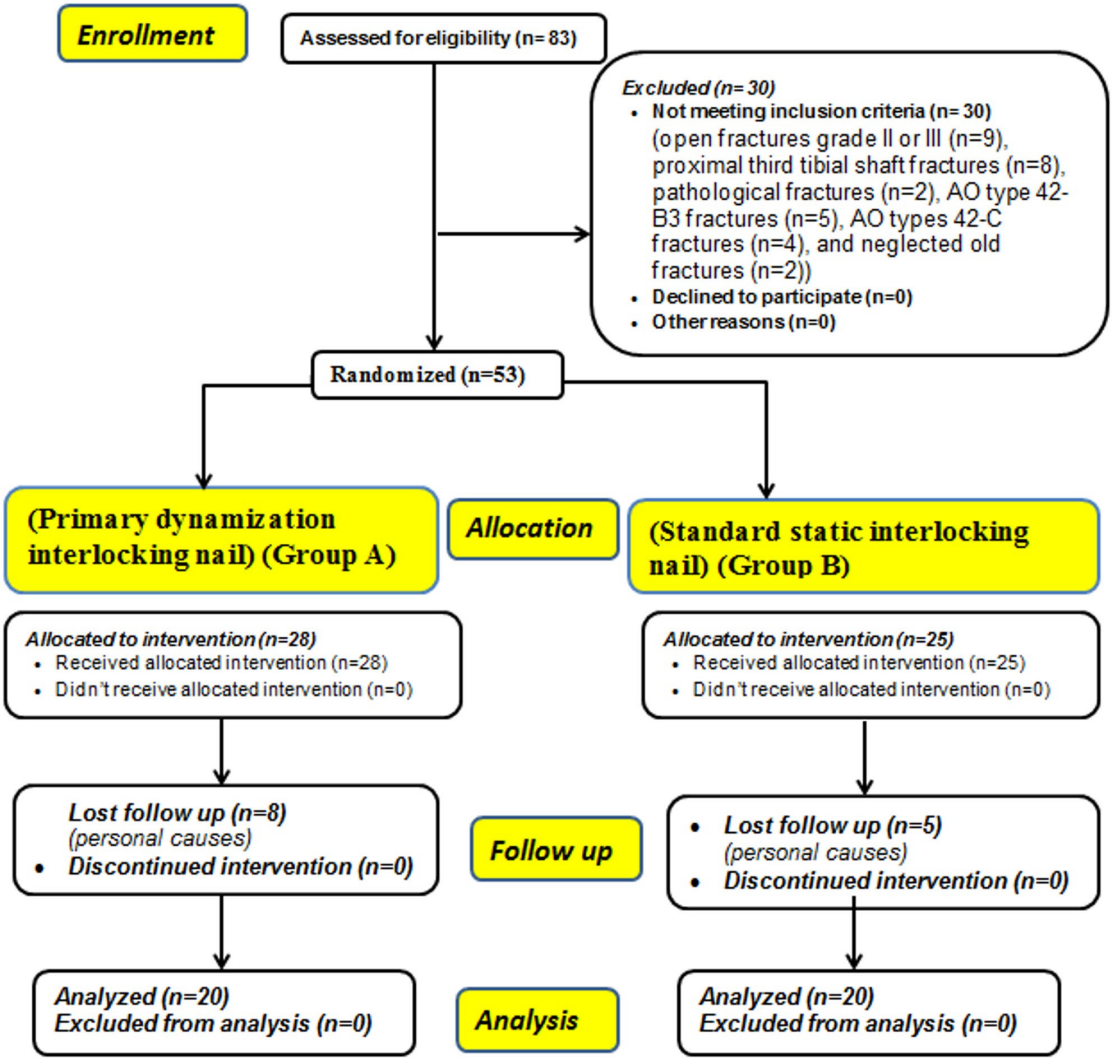
Consolidated standards of reporting trials (CONSORT) flow chart of the patients’ enrollment

During the study period, 83 patients were evaluated for eligibility; 30 patients didn’t meet the inclusion criteria (open fractures grade II or III (*n* = 9), proximal third tibial shaft fractures (*n* = 8), pathological fractures (*n* = 2), AO type 42-B3 fractures (*n* = 5), AO types 42-C fractures (*n* = 4), and neglected old fractures (*n* = 2)), and only 53 patients were eligible for inclusion (28 patients were treated by primary dynamization ILN (group A) and 25 by Standard static ILN (group B)). 13 patients were lost during follow up due to personal causes; (in group A (*n* = 8), and in group B (*n* = 5)) leaving 40 patients for final analysis who were completed at least one year follow up [20 patients in each group], details of allocation are shown in Fig. 4.

The mechanism of injury in the vast majority of the patients was road traffic accident. No differences were founded between the two groups regarding age, sex, side affected, mechanism of injury, AO/OTA types of fractures, level of tibial fractures, associated fibular fractures, smoking, time elapsed between trauma and surgery, hospital stays, or follow up duration.

Duration of surgery in group A (Primary dynamization ILN) was significantly shorter at 66.25 ± 4.83 (60–75) min compared to group B (Standard static ILN) at 75 ± 5.38 (65–85) min, *(P =*.044*)* (Table 1).

**Table 1:** Demographics and clinical characteristics of the study groups

Characteristics	Primary dynamization ILN (Group A)			Standard static ILN (Group B)	*P*-value
Number of patients	20			20	
Age at time of operation (years)					
Mean ± SD (Range)	33.25 ± 8.26 (18–48)			31.45 ± 8.24(19–47)	.074 (NS)
Sex					
Males, no (%)	17 (85%)			15 (75%)	.429 (NS)
Females, no (%)	3 (15%)			5 (25%)	
Side affected					
Right, no (%)		11 (55%)		8 (40%)	.342 (NS)
Left, no (%)		9 (45%)		12 (60%)	
Mechanism of injury					
Road traffic accident, no (%)		19 (95%)		20 (100%)	.311 (NS)
Falling from height, no (%)		1 (5%)		0 (0%)	
AO/OTA classification of fractures					
42-A1		5(25%)		8(40%)	.056 (NS)
42-A2		7(35%)		8(40%)	
42-A3		8(40%)		1(5%)	
42-B1		0 (0%)		2(10%)	
42-B2		0 (0%)		1(5%)	
Classification of fractures according to soft tissue condition					
Closed fractures, no (%)			13 (65%)	16(80%)	.144 (NS)
Open fractures (grade I), no (%)			7 (35%)	4(20%)	
Level of tibial fractures					
Mid shaft, no (%)		16(80%)		15(75%)	.705 (NS)
Distal shaft, no (%)		4(20%)		5(25%)	
Associated fibula fractures					
Absent, no (%)	4 (20%)			7 (35%)	.288 (NS)
Present, no (%)	16 (80%)			13 (65%)	
Smoking					
Non-smokers, no (%)	13 (65%)			12 (60%)	.744 (NS)
Smokers, no (%)	7 (35%)			8 (40%)	
Time elapsed between trauma and surgery (days)					
Mean ± SD (Range)	4 ± 2.5 (1–10)			3.07 ± 3.45 (1–10)	.234 (NS)
Duration of surgery (min)					
Mean ± SD (Range)	66.25 ± 4.83 (60–75)			75 ± 5.38 (65–85)	.044 (S)
Hospital stays (days)					
Mean ± SD (Range)	1.6 ± 0.5 (1–2)			1.7 ± 0.6 (1–3)	.737 (NS)
Follow up period (months)					
Mean ± SD (Range)	12.25 ± 2.43 (12–18)			12.34 ± 2.54 (12–18)	.434 (NS)

A Radiographic outcomes
  All fractures healed before six months without implant failure or malunion. The mean time needed for complete radiographic union was significantly shorter in group A (Primary dynamization ILN) at 14±1.86 (range, 12–18) weeks compared to group B (Standard static ILN) at 16±1.86 (range, 14–20) weeks, (P <.001) (Table 2).

**Table 2:** Radiographic and functional outcomes of the study groups

Outcomes		Primary dynamization ILN (Group A)		Standard static ILN (Group B)	*P*-value
Time of radiographic complete union (weeks)					
Mean ± SD (Range)		14 ± 1.86 (12–18)		16 ± 1.86 (14–20)	< .001 (S)
Union rate, no (%)					
		20/20 (100%)		20/20 (100%)	> .999 (NS)
Time of full weight-bearing (weeks)					
Mean ± SD (Range)	6.75 ± 1.12 (6–10)		8.55 ± 2.16 (6–14)		.007 (S)
Bostman knee score at final follow up (points)					
Mean ± SD (Range)		28.05 ± 2.33 (21–30)		26.1 ± 2.65 (21–30)	.016 (S)
Excellent, no (%)		16 (80%)		14 (70%)	.072 (NS)
Good, no (%)		4 (20%)		6 (30%)	
Poor, no (%)		0 (0%)		0 (0%)	
AOFAS ankle-hindfoot score at final follow up (points)					
Mean ± SD (Range)		94.6 ± 5.3 (75–100)		93 ± 4.6 (75–100)	.091 (NS)
Excellent, no (%)		15 (75%)		14 (70%)	.085 (NS)
Good, no (%)		5 (25%)		6 (30%)	
Fair, no (%)		0 (0%)		0 (0%)	
Poor, no (%)		0 (0%)		0 (0%)	
VAS score for pain (points)					
Mean ± SD (Range)		0.75 ± 1.45 (0–5)		0.9 ± 1.84 (0–4)	.514 (NS)
B Functional outcomes
  The time of full weight bearing was significantly earlier in group A (Primary dynamization ILN) at 6.75±1.12 (range, 6–10) weeks compared to group B (Standard static ILN) at 8.55±2.16 (range, 6–14) weeks, (P =.007).
  At one year follow up; full ROM of knee and ankle joints were obtained in both groups. No significant difference were found between both groups as regard of AOFAS ankle-hindfoot score, and VAS for pain, (P =.091 &.514 respectively). Bostman knee score was significantly higher in group A (Primary dynamization ILN) at 28.05±2.33 (range, 21–30) points compared to group B (Standard static ILN) at 26.1±2.65 (21–30) points, (P =.016). There were 16 (80%) patients with excellent scores and 4 (20%) patients with good scores in group A, whereas there were 14 (70%) patients with excellent scores and 6 (30%) patients with good scores in group B, without poor scores in the two groups, (P=.072) (Table 2).
C Complications
  No significant difference was found between two groups regarding anterior knee pain, superficial or deep infections (Table 3).

**Table 3:** Complications of the study groups

Outcomes	Primary dynamization ILN (Group A)	Standard static ILN (Group B)	*P*-value
Superficial infections	1(5%)	0 (0%)	.572 (NS)
Deep infections	1(5%)	2(10%)	
Anterior knee pain			
Absent	17 (85%)	15 (75%)	.073 (NS)
Mild	2 (10%)	3 (15%)	
Moderate	1 (5%)	2 (10%)
D Factors affecting the outcomes
  Factors associated with excellent outcomes in primary dynamization ILN (group A) were; mid-shaft tibial fractures, associated fibular fractures, and less severity of fracture type according to AO/OTA classification (p<.001,.013 &.042 respectively). However, only closed fractures were associated with excellent outcomes in standard static ILN (group B), (p=.027). Non-smokers had excellent outcomes in both groups, (p=.011 &.049 respectively) (Table 4).

**Table 4:** Factors affecting functional outcomes according to Bostman score in the two study groups

				Primary dynamization ILN (Group A)			Standard static ILN (Group B)		
				**Bostman knee score**		**p**-**value**	**Bostman knee score**		**p**-**value**
				Excellent (*n* = 16)	**Good** (*n* = 4)		**Excellent** (*n* = 14)	**Good** (*n* = 6)	
Age (years)^a^				28 ± 6.26	37 ± 4.6	.074^c^	33 ± 3.24	38.2 ± 5.5	.083^c^
Sex^b^	Male			15 (93.8%)	2 (50%)	.067^d^	10 (71.4%)	5 (83.3%)	.072^d^
	Female			1 (6.2%)	2 (50%)		4 (28.6%)	1 (16.7%)	
Smoking^b^	Non-smokers			12 (75%)	1 (25%)	**.011**^**d**^	10 (71.4%)	2 (33.3%)	**.049**^**d**^
	Smokers			4 (25%)	3 (75%)		4 (28.6%)	4 (66.7%)	
Level of tibial fractures^b^		Mid shaft		15 (93.8%)	1 (25%)	**< .001**^**d**^	12 (85.7%)	3 (50%)	.085^d^
		Distal shaft		1 (6.2%)	3 (75%)		2 (14.3%)	3 (50%)	
Associated fibula fractures^b^		Absent		2 (12.5%)	2 (50%)	**.013**^**d**^	5 (35.7%)	2 (33.3%)	.236^d^
		Present		14 (87.5%)	2 (50%)		9 (64.3%)	4 (66.7%)	
Classification of fractures according to soft tissue condition^b^		Closed fractures		10 (62.5%)	3 (75%)	.141^d^	12 (85.7%)	4 (66.7%)	**.027**^**d**^
		Open fractures (grade I)		6 (37.5%)	1 (25%)		2 (14.3%)	2 (33.3%)	
AO/OTA classification of fractures^b^		42-A1 5		4 (25%)	1 (25%)	**.042**^**d**^	6 (42.8%)	2 (33.3%)	.424^d^
		42-A2 7		5 (31.25%)	2 (50%)		7 (50%)	1 (16.7%)	
		42-A3 8		7 (43.75%)	1 (25%)		1 (7.2%)	0	
		42-B1		0	0		0	2 (33.3%)	
		42-B2		0	0		0	1 (16.7%)	
Time of radiographic complete union (weeks)^a^				12 ± 0.5	14 ± 0.8	.232^c^	14 ± 0.3	16 ± 0.7	.275^c^
Time of full weight bearing (weeks)^a^				6 ± 0.68	8 ± 1.3	.623^c^	8 ± 0.46	10 ± 0.7	.521^c^

^a^Data are presented as mean ± standard deviation

^b^Data are presented as No (%)

^c^One-way ANOVA

^d^Chi-square test

## Discussion

In the current RCT study, we have introduced a novel surgical technique of primary dynamization of ILN tibia without use of proximal locking screws in treatment of middle or distal thirds tibial shaft fractures in adults. Moreover, we compared the functional and radiological outcomes of this novel surgical technique to the standard static ILN in treatment of middle or distal thirds tibial shaft fractures in adults.

After a follow up period of 12.25 ± 2.43 months for primary dynamization ILN (group A), and 12.34 ± 2.54 months for standard static ILN (group B), our hypothesis was confirmed as the primary dynamization of ILN tibia without use of proximal locking screws demonstrated superior outcomes in comparison to standard static ILN tibia regarding shorter duration of surgery, shorter time to complete union, better functional outcomes and lower complication rates (*p* =.044, < .001, =.016, = .572& = .073 respectively).

Primary dynamization of ILN tibia without use of proximal locking screws permits inter-fragmentary compression and maximizes the bone fragments contact at the fracture site, leading to rapid healing and callus formation. However, this technique is inappropriate for comminuted and segmental fractures as dynamization leads to excessive compression, bone resorption, osteonecrosis at the fracture site, limb shortening, non-unions, and pain.

Adequate rotational stability of primary dynamization ILN tibia without proximal locking screws was obtained by many factors; (1) use of the largest possible nail diameter to allow press-fit impaction within the medullary cavity, (2) the proximal nail bend (Herzog angle) prevents rotation, (3) choice of specific types of middle and distal thirds tibial shaft fractures (AO types of 42-A, 42-B1 or 42-B2), and (4) exclusion of proximal third shaft fractures.

Saruhan et al. [24] retrospectively reviewed 30 patients who performed locked (*n* = 15) or unlocked intramedullary nails (*n* = 15) for tibial shaft fractures between December 2006 and July 2011. They found that the demographic and clinical data as sex, age, mechanism of injury, fracture location and morphology, open fracture types, and time to discharge were similar at both groups, and no statistically significant difference in functional results according to Johner-Wruhs criteria between both groups. They concluded that although primary treatment for tibial shaft fractures is locked intramedullary nailing, unlocked nailing would take place for selected cases.

Hernández-Vaquero et al. [25] retrospectively reviewed closed or type I open tibial diaphyseal fractures (AO types A and B) treated with dynamic locking nailing with early full weight-bearing (32 cases) or static locking nailing with delayed weight bearing until bone union (35 cases), and found that the mean time to union was 21 weeks in the dynamic locking and 26 weeks in the static locking (*p* =.051). They concluded that dynamic intramedullary nailing is safe and should be encouraged for these fracture patterns.

In the current study; partial weight-bearing was started immediate post-operatively as soon as the patient could tolerate, whereas full weight-bearing was delayed until appearance of signs of bony union which was significantly earlier in group A (Primary dynamization ILN) at 6.75 ± 1.12 weeks compared to group B (Standard static ILN) at 8.55 ± 2.16 weeks, *(P =*.007*)*. This is consistent with the protocol of Hernández-Vaquero et al. [25]. Also, Gross et al. [26], examined the possible benefits and risks of weight-bearing after ILN of unstable tibial shaft fractures, and they found that no statistical difference was observed in time to union between groups (immediate weight-bearing-as-tolerated (WBAT) = 22.1 ± 11.7 weeks vs. non-weight-bearing for the first 6 postoperative weeks (NWB) = 21.3 ± 9.9 weeks; *P* =.76). They concluded that immediate weight-bearing after ILN tibial shaft fractures was safe, not associated with an increase in adverse events or complications, and patients should be allowed to weight bear as tolerated after ILN of AO subtype 42-A and 42-B tibial shaft fractures.

The robustness and reliability of this study was accomplished by many points of strength. First, the study design is open-labeled RCT which is a strong research methodology that reduces bias and assures reliable outcomes. Second, it introduced a novel surgical technique of primary dynamization ILN tibia without use of proximal locking screws in treatment of middle or distal thirds tibial shaft fractures in adults and confirmed its superior functional and radiographic outcomes compared to standard static ILN. Therefore, it provides helpful insights for the best treatment methods of these fractures and reduces the socioeconomic burdens of tibial shaft non-unions or delayed unions.

The main limitations of this study are; lack biomechanical experiment, lack of measurement of the difference in fluoroscopy/radiation exposure times between the two groups, short follow-up duration, small sample size, and being single center. In addition, primary dynamization ILN without use of proximal locking screws is only feasible for middle or distal thirds tibial shaft fractures of AO types 42-A, 42-B1, or 42-B2 which limit the generalizability of findings.

Larger, multi-center studies with longer follow up are advised in the future studies for validation and expansion of these findings.

## Conclusion

Both techniques achieved complete bony union and pain relief with minor complications. Primary dynamization of ILN with no proximal locking screws for the treatment of middle or distal thirds tibial shaft fractures in adults had potential advantages over standard static ILN including; shorter duration of surgery, faster radiographic complete union, rapid full weight-bearing, and better functional outcomes.

Proper selection of patients, fracture types, and strict follow up are essential for success of primary dynamization ILN without use of proximal locking screws.

## Supplementary Information

Supplementary Material 1.

## Data Availability

The datasets generated and/or analyzed during the current study are available from the corresponding author on reasonable request.

## Abbreviations

AP: Antero-posterior
ILN: Interlocking nail
RCT: Randomized controlled trial
VAS: Visual Analogue Scale
AOFAS: American Orthopaedic Foot and Ankle Society
mRUST: Modified radiographic union scale for tibial fractures
WBAT: Weight-bearing-as-tolerated
NWB: Non-weight-bearing
